# Biocatalysis for the application of CO_2_ as a chemical feedstock

**DOI:** 10.3762/bjoc.11.259

**Published:** 2015-12-01

**Authors:** Apostolos Alissandratos, Christopher J Easton

**Affiliations:** 1 Research School of Chemistry, Australian National University, Canberra ACT 2601, Australia

**Keywords:** biocatalysis, carboxylase, CO_2_ transformation, formate dehydrogenase, RuBisCO

## Abstract

Biocatalysts, capable of efficiently transforming CO_2_ into other more reduced forms of carbon, offer sustainable alternatives to current oxidative technologies that rely on diminishing natural fossil-fuel deposits. Enzymes that catalyse CO_2_ fixation steps in carbon assimilation pathways are promising catalysts for the sustainable transformation of this safe and renewable feedstock into central metabolites. These may be further converted into a wide range of fuels and commodity chemicals, through the multitude of known enzymatic reactions. The required reducing equivalents for the net carbon reductions may be drawn from solar energy, electricity or chemical oxidation, and delivered in vitro or through cellular mechanisms, while enzyme catalysis lowers the activation barriers of the CO_2_ transformations to make them more energy efficient. The development of technologies that treat CO_2_-transforming enzymes and other cellular components as modules that may be assembled into synthetic reaction circuits will facilitate the use of CO_2_ as a renewable chemical feedstock, greatly enabling a sustainable carbon bio-economy.

## Introduction

Depletion of fossil-fuel feedstocks and pollution resulting from their unsustainable processing and use constitute challenging global issues [1–2]. Catalysis has an important role to play in addressing these challenges through the generation of fuels and commodity chemicals from renewable sources in a sustainable manner [3]. In this context, CO_2_ has become a compound of key interest as it is one of the main contributors to fossil-fuel pollution [4–5]. As a result, decreasing CO_2_ emissions and CO_2_ sequestration technologies are subjects of intense research. In addition, CO_2_ may hold an even more important role in a sustainable future, as a readily available and renewable material that may be utilised as an alternative feedstock for the production of many of the chemicals we have come to rely on [6–11]. Chemical processes that employ CO_2_ as a synthon for the production of commodity chemicals may form the basis of a sustainable carbon economy.

The benefits notwhithstanding, chemical conversion of CO_2_ into other forms of carbon remains challenging because the transformations typically have high activation barriers and are therefore very energy intensive [12]. Catalysis will therefore play a critical role in the development of viable solutions for the transformation of CO_2_. Biocatalysts are very likely to contribute towards this end due to their ability to efficiently catalyse processes under mild conditions with limited byproduct formation [13–14]. These catalysts have been developed by nature to utilise diverse substrates including simple compounds such as CO_2_. Indeed, life itself depends on the ability of autotrophic organisms to convert CO_2_ into other materials, and these are therefore a valuable source of the required biocatalysts.

The development of methodologies for expression, characterisation, engineering and optimisation of CO_2_-transforming enzymes will form the basis of any future biotechnology that aims to use CO_2_ as a feedstock for the generation of other materials. Here we provide an overview of the biocatalysts that have already been applied to relevant technologies and are set to play an important role in future bioprocesses for the transformation of CO_2_ into fuels and commodity chemicals. As well as reviewing applications of these biocatalysts, we highlight the chemical, biochemical and biological contexts in which they operate, the understanding of which is critical for effective application. As commodity chemicals contain carbon at lower oxidation states than CO_2_, only enzymes that involve CO_2_ reduction will be covered here and not carbonic anhydrase for the conversion to HCO_3_^−^, which is extensively covered in other reviews on carbon-capture technology [15].

## Review

### Biotechnological transformation of CO_2_

Synthesis of commercial materials through the biological transformation of CO_2_ is the basis of all agriculture. Through the cultivation of crops, CO_2_ is converted into more useful forms of carbon, such as starch and lignocellulosic materials. In turn, these materials have been employed as carbon sources for fermentative processes, and more recently in first and second generation biofuel production processes. In this way, the carbon fixed by plants (biomass) is further transformed into a wide array of products through microbial processing [16]. Genetically engineered plants and algae have been employed to divert carbon flux in planta towards other metabolic products of interest, as an alternative to microbial processes [17–18]. Yet another alternative approach is to directly fix the CO_2_ with microorganisms, circumventing the intermediacy of crop derived biomass [19–20]. This can be done with autotrophic microbes, though these are generally poorly understood, and the genetic tools required to divert carbon flux towards useful products are still under-developed with these species. Alternatively, as discussed in detail below, well understood microbes for which genetic modification methodologies are widely available, such as *E. coli*, have been used as hosts for heterologous CO_2_ fixation reactions [21], that may then be coupled to an extensive array of metabolic pathways for the delivery of target compounds.

### Biological strategies to increase CO_2_ reactivity

#### Energetic demand of CO_2_ transformation

Most of the carbon associated with fossil-fuel based technologies will eventually be converted to CO_2_ through combustion or oxidative degradation [12,19,22], because this is the most oxidised and stable state of carbon (+4). Converting CO_2_ into other more reduced forms of carbon, as found in organic commodity chemicals, requires large energy inputs. As a result, there are only a limited number of examples of industrial chemical processes which use CO_2_ as feedstock, and those that do, such as the Bosch–Meiser process [7], are very energy intensive.

**Figure 1:**
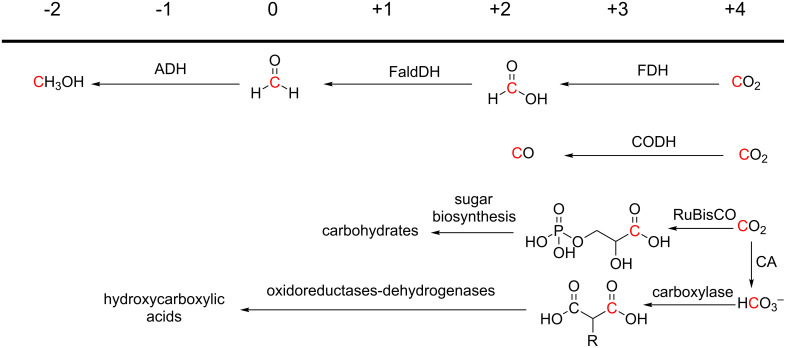
Biocatalytic routes for conversion of CO_2_ into compounds with carbon in the reduced oxidation states indicated at the top. FDH: formate dehydrogenase, FaldDH: formaldehyde dehydrogenase, ADH: alcohol dehydrogenase, CODH: carbon monoxide dehydrogenase, RuBisCO: ribulose-1,5-bisphosphate carboxylase oxygenase, CA: carbonic anhydrase, R: H, CH_3_.

Associated with the dependence of autotrophic organisms on CO_2_ as a carbon source, biological systems have developed various strategies to avoid energy constraints, and as a result there are several metabolic pathways for reductive transformation of CO_2_ (Figure 1) [23–24]. Generally, such processes are driven by coupling the CO_2_ transformations with oxidations that generate reducing equivalents, sometimes in conjunction with the hydrolysis of phosphoanhydride bonds [25–27]. For reductases or dehydrogenases operating in reverse, the electrons required to reduce CO_2_ are provided through oxidation of reduced forms of redox cofactors, either directly or through electron driving protein mediation (NAD(P)H or equivalents). For a number of carboxylases, phosphoanhydride bonds in ATP are hydrolysed to drive CO_2_ transformation through various molecular mechanisms. For example, biotin carboxylase catalysed reactions proceed through electrophilic activation of CO_2_ to carboxyphosphate to facilitate an attack by a nucleophile [28].

In all known natural CO_2_ fixation pathways, ATP and NADH or their equivalents are consumed in order to generate the intermediates that may feed into central metabolism [23]. This consumption is used as a measure of pathway efficiency for CO_2_-fixation, and pathways are considered most efficient when it is minimised [25,27]. By balancing thermodynamic feasibililty and a low requirement in NADH and ATP or equivalents, Milo and coworkers [25] were able to computationally predict the most efficient synthetic CO_2_ fixation pathways, using all known natural enzymes.

#### Aqueous solubility and hydration of CO_2_

**Figure 2:**
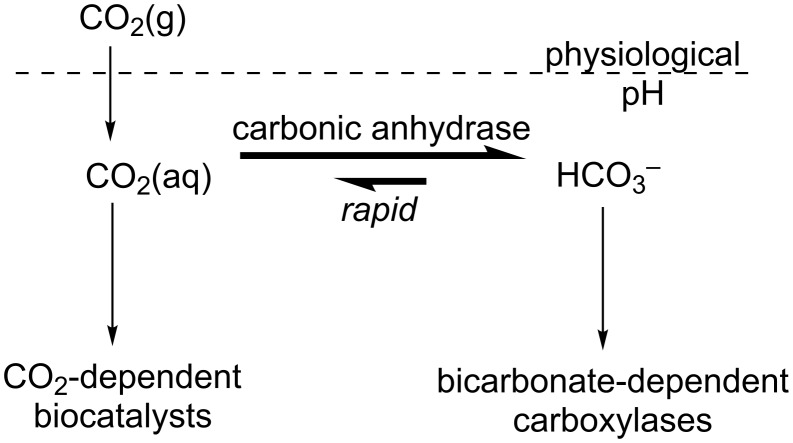
Carbonic anhydrase-catalysed rapid interconversion of CO_2_ and HCO_3_^−^ in living systems.

A particular limitation for aqueous CO_2_ transformations stems from the low concentration of dissolved CO_2_ at saturation. At physiological pH, CO_2_ is hydrated and exists predominantly as the bicarbonate anion (HCO_3_^−^) [29]. Within cells, CO_2_ and HCO_3_^−^ rapidly interconvert through catalysis by carbonic anhydrase, the archetypal super-enzyme for which catalytic rates reach the limits of diffusion [30–31]. CO_2_ consumed by enzymes is therefore efficiently replenished through rapid HCO_3_^−^ dehydration (Figure 2). Living organisms have developed various mechanisms to increase the effective concentration of CO_2_, ranging from the use of carboxylated cofactors [28,32] to complex extended metabolic pathways in C_4_ and CAM plants [17,33–34] and substrate channelling. In addition, a number of enzymes accept HCO_3_^−^ as a substrate, which is converted to CO_2_ close to the active site before the reductive step [26,28].

In photoautotrophic bacteria (cyanobacteria) micro-compartmentalisation of the CO_2_-fixing reactions increases reaction rates [35–36]. The bacterial micro-compartments, called carboxysomes, are highly elaborate proteinic structures that usually also incorporate carbonic anhydrase [36–37]. Carboxysomes have been the subject of studies on increasing the efficiency of C_3_ carbon fixation in plants [38–40]. The recent production of a transgenic tobacco plant, expressing bacterial carboxysome proteins and able to photosynthesise at an increased rate, was a significant breakthrough in this field [39]. Carboxysomal proteins have also been expressed in *E. coli* yielding a highly organised structure [41]. Use of carboxysomes for micro-compartmentalisation of CO_2_ biotransformation may therefore become a viable strategy in a range of synthetic biology applications, because not only CO_2_-transforming enzymes, but also the cohort of supporting cellular equipment and mechanisms that living systems employ, may be used to drive these processes.

### Sources of CO_2_ transforming enzymes

#### Emergence of CO_2_ transforming enzymes

Autotrophic enzymes have evolved to promote and control CO_2_ fixation and are an obvious starting point for the biotechnological transformation of CO_2_ [42].

To understand the properties and distribution of these CO_2_-assimilating enzymes, it is important to consider the geochemical context in which they have evolved as there appears to be a strong link with atmospheric concentrations of CO_2_. The environment from which life emerged is thought to have been anoxic with high concentrations of CO_2_ [43]. In this environment, the first CO_2_-fixing enzymes evolved to take advantage of the most readily available carbon source. Through the action of these enzymes and geological processes for CO_2_ sequestration, CO_2_ concentrations steadily decreased, leading to average atmospheric concentrations of 200 ppm over the last 400,000 years [44]. During this time, oxygen levels steadily increased through the action of photosynthetic organisms that oxidise water to produce molecular oxygen [43]. Consequently many CO_2_-assimiliating enzymes evolved to be strictly anaerobic, and are limited to specific environments, while others tolerate O_2_ [45]. As a result, the environmental [CO_2_]/[O_2_] ratio is an important effector of enzymatic properties.

#### RuBisCO and the Calvin cycle

**Scheme 1:**
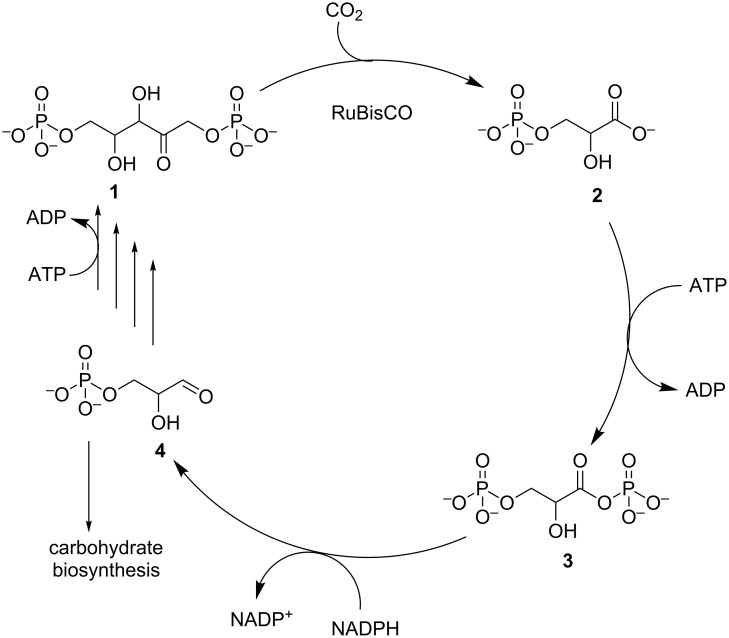
The Calvin cycle for fixation of CO_2_ with RuBisCO.

For many years, the Calvin cycle for C_3_ carbon fixation was thought to be the only important biological process for CO_2_ assimilation, as a result of its prevalence in our immediate environment. It is found in photosynthetic organisms, predominantly in plants on land and algae in water, and photosynthetic prokaryotes (cyanobacteria). This carbon fixation pathway forms part of photosynthesis and the required reducing equivalents are generated through electron gradients initiated by photons and generated through the splitting of water [46]. However, a number of autotrophic bacteria fix carbon through the Calvin cycle with electrons generated through oxidation of inorganic chemicals (chemoautotrophs) [47]. As detailed in Scheme 1, the carbon fixation step entails the carboxylation of ribulose-1,5-bisphosphate (**1**), generating two equivalents of 3-phosphoglycerate (**2**) and is catalysed by ribulose-1,5-bisphosphate carboxylase oxygenase (RuBisCO). The glycerate **2** is subsequently phosphorylated with ATP for the production of 1,3-bisphosphoglycerate (**3**), which is in turn reduced with NADPH to 3-phosphoglyceraldehyde (**4**). For every six equivalents of the aldehyde **4**, one is diverted to carbohydrate biosynthesis, while the other five are used to produce the RuBisCO substrate **1**.

A property of RuBisCO with great implications is that it may also accept O_2_ instead of CO_2_ as an electrophile in the addition step, thus catalysing a counter-productive reaction, which reduces the photosynthetic output of plants using the Calvin cycle by 25% [48]. In the O_2_-rich environments in which it operates, this property makes RuBisCO a particularly inefficient biocatalyst and a major bottleneck to C_3_ carbon fixation. Through evolution, RuBisCO has adapted to rising oxygen concentrations by developing higher specifities for CO_2_ at the expense of catalytic turnover, making it a particularly slow enzyme [49]. As a result, evolutionary bias from limited nutrient availability has driven some plants to develop more elaborate carbon assimilation mechanisms (C_4_ and CAM plants) [48]. These involve an initial temporary carbon fixation step with phosphoenolpyruvate carboxylase (PEPC), followed by transport and release as CO_2_ in the vicinity of RuBisCO within cellular compartments with low O_2_ concentrations [48]. RuBisCO variants from these plants display higher turnover rates, and lower specificities for CO_2_ over O_2_. This apparent trade-off between CO_2_ specificity and catalytic activity greatly influences efforts towards RuBisCO biotechnological applications.

The Calvin cycle is not the only carbon fixation pathway, and at least five alternative pathways have been elucidated in recent years [24]. It is now thought that some of these alternative pathways contribute significantly to the global carbon cycle, particularly with regard to the oceanic section [50–51]. This is due to the extensive global distribution of many oceanic chemolithoautotrophic organisms, and the estimated carbon fixation in deep-sea hydrothermal vents, the meso- and bathy-pelagic ocean, and in oxygen-deficiency zones [50].

#### Reductive tricarboxylic acid cycle

The tricarboxylic acid (TCA) cycle is used by all aerobic organisms to generate NADH through the oxidation of small organic metabolites. For pyruvate (**11**), isocitrate (**7**) and 2-oxoglutarate (**6**), oxidation occurs together with a decarboxylation. In some autotrophs this pathway is known to operate in the reverse (reductive) direction resulting in CO_2_ fixation through carboxylation [52]. Autotrophic fixation through the reductive TCA cycle was first described by Arnon and Buchanan [53], and hence is also referred to as the Arnon–Buchanan cycle. It is considered the most efficient CO_2_ fixation pathway as it requires the lowest amount of reducing equivalents per carbon fixed [23,26]. This is mainly due to the fact that CO_2_ fixation occurs through three efficient reductive carboxylations, of which two are coupled to oxidation of the low-potential electron donor ferredoxin [26], with a requirement for strict anaerobicity, thus limiting the distribution of the reductive TCA cycle.

**Scheme 2:**
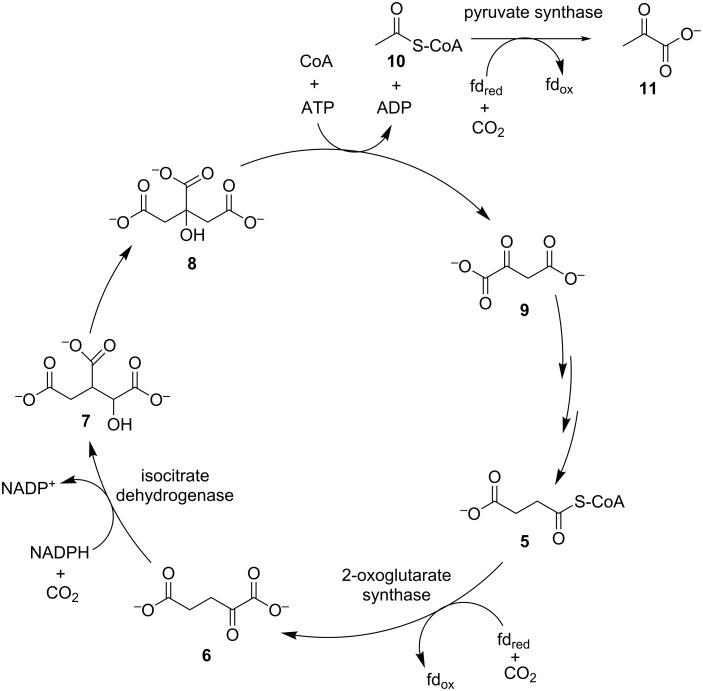
The reductive TCA cycle with CO_2_ fixation enzymes designated.

As detailed in Scheme 2, the reductive TCA cycle contains three CO_2_ fixation steps [24]. Succinyl-CoA (**5**) is carboxylated by ferredoxin-dependent 2-oxoglutarate synthase to produce 2-oxoglutarate (**6**), which is subsequently transformed to isocitrate (**7**) through a second CO_2_ fixation catalysed by isocitrate dehydrogenase. Isocitrate (**7**) is concominantly isomerised to citrate (**8**) and lysed to oxaloacetate (**9**) which remains in the cycle and regenerates succinyl-CoA through three catalytic steps, and acetyl-CoA (**10**) which enters central metabolism through a third CO_2_-fixation step, carried out by ferredoxin-dependent pyruvate synthase to produce pyruvate (**11**). The carboxylating enzymes are mechanistically complex and highly adapted to the cellular conditions in which they operate, and as a result there has been little development of their use in synthetic processes.

#### Wood–Ljungdahl pathway

**Scheme 3:**
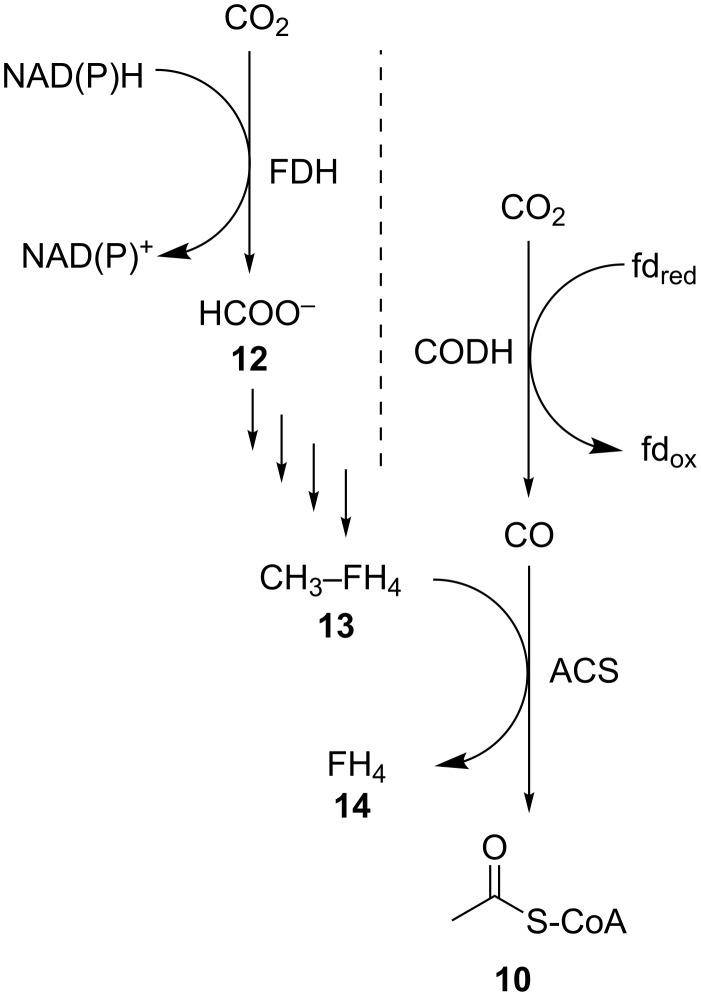
The Wood–Ljungdahl pathway for generation of acetyl-CoA through reduction of CO_2_ to formate and CO. FDH: formate dehydrogenase, CODH: CO dehydrogenase, ACS: acetyl-CoA synthase, FH_4_: tetrahydrofolate.

The Wood–Ljungdahl pathway, or reductive acetyl-CoA pathway, is used by acetogenic bacteria to reduce CO_2_ to either formate with formate dehydrogenase (FDH) or CO with CO dehydrogenase (CODH) [54–55]. As presented in Scheme 3, these initial steps of two separate branches of the pathway meet to produce a unit of acetyl-CoA (**10**) which is then incorporated into central metabolic processes [56–59]. FDHs are widely distributed enzymes, discussed in more detail below. The formate (**12**) produced through FDH activity is incorporated onto a tetrahydrofolate (**14**) and reduced to an activated methyl group (**13**), which is then utilised as a substrate by acetyl-CoA synthase together with the CO produced by CODH. The acetyl-CoA synthase forms a complex with CODH, to channel CO through a molecular tunnel [60]. This enzyme has been the focus of much interest due to its unusual reactivity, however, it remains poorly understood [61].

Formate dehydrogenases are an extremely heterogeneous enzyme family, most commonly found to physiologically catalyse formate oxidation and release of CO_2_. Autotrophic acetogen FDHs are usually bound to metallo-pterin cofactors, with either a Mo or W centre [55,62–63], coordinated to a SeCys or Cys ligand. These features are not limited to acetogenic FDHs, and Mo and W FDHs are broadly distributed throughout the bacterial kingdom [63–68]. In addition, various types of Fe–S clusters are observed in FDHs, through which electrons are transported to other protein domains or to other oxidoreductases altogether [63–64,68]. Due to the presence of oxidisable cofactors, metallo-FDHs are most commonly found in anaerobic organisms. Another large family of FDHs do not contain metal cofactors, and catalyse a direct hydride transfer from formate to a nicotinamide cofactor [69]. They are commonly found in aerobic species, are generally robust and amenable to recombinant expression, but have high catalytic preferences for formate oxidation to CO_2_.

#### Acyl-CoA pathways

**Scheme 4:**
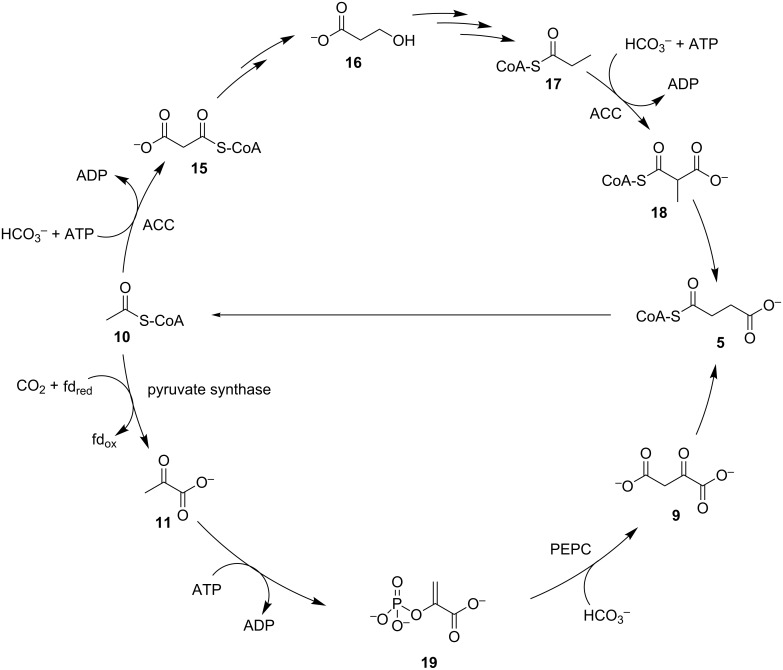
The acyl-CoA carboxylase pathways for autotrophic CO_2_ fixation. ACC: acetyl-CoA/propionyl-CoA carboxylase, PEPC: phosphoenolpyruvate carboxylase.

A number of recently elucidated cyclic pathways that exist primarily in archaea initiate through the fixation of CO_2_ onto acetyl-CoA (**10**) [51,70], and end with the generation of two equivalents of the starting substrate **10** (Scheme 4). One equivalent of the CoA thioester **10** is fed to central metabolism while the other is used in a subsequent cycle. As seen in Scheme 4, acetyl-CoA (**10**) is carboxylated by a bifunctional acetyl-CoA/propionyl-CoA carboxylase to malonyl-CoA (**15**) with HCO_3_^−^ and hydrolysis of ATP. The malonate **15** is reduced to 3-hydroxypropionate (**16**), in two steps catalysed by NAD(P)H dependent dehydrogenases. Later in the pathway, propionyl-CoA (**17**) is the substrate for a second carboxylation with HCO_3_^−^ to methylmalonyl-CoA (**18**), performed by the same ATP-dependent bifunctional carboxylase that carries out the first step [71–72]. Succinyl-CoA (**5**) is formed through isomerisation and recycled into two equivalents of acetyl-CoA (**10**). This route is encountered in two separate pathways, namely the 3-hydroxypropionate/4-hydroxybutyrate cycle and the 3-hydroxypropionate bicycle. An alternative CO_2_ fixation route is found in the dicarboxylate/4-hydroxybutyrate cycle [73]. Here acetyl-CoA (**10**) is initially reductively carboxylated to pyruvate (**11**), as in the reductive TCA cycle. The pyruvate **11** is phosphorylated with ATP to generate phosphoenolpyruvate (**19**), followed by a second carboxylation with HCO_3_^−^ to oxaloacetate by PEPC.

#### Non-autotrophic CO_2_ fixation

A large number of enzymes use CO_2_ (or HCO_3_^−^) as a substrate without taking part in autotrophic pathways [26]. These are predominantly found in assimilatory pathways where small organic molecules are used as carbon sources, and anaplerosis through which intermediate metabolites in central pathways (e.g., the TCA cycle) are replenished.

These enzymes also represent interesting targets for use in CO_2_ transforming processes, particularly when involved in the production of TCA cycle dicarboxylates that constitute target platform chemicals, as in the cases of pyruvate carboxylase and PEPC. Enzymes that catalyse CO_2_ fixation in autotrophic pathways are also found in non-autotrophic pathways operating either in the same direction (PEPC), or in the reverse direction for CO_2_ production (FDH). However, these enzymes are still suitable targets and have been used in vitro for CO_2_ fixation. Finally degradative pathways contain enzymes capable of working in both carboxylating or decarboxylating direction depending on reaction conditions [12]. These, have also attracted some attention as a source for relevant biocatalysts.

### Biotechnological CO_2_ transformation

CO_2_-transforming enzymes sourced from natural metabolic pathways have been utilised in biotechnological applications for the conversion of CO_2_, through either direct reduction of CO_2_ or carboxylation of another substrate.

#### CO_2_ transformation with RuBisCO

As the most well studied and best characterised autotrophic CO_2_-fixation enyzme, RuBisCO has received much attention for application in biotechnology for CO_2_-fixation, particularly using engineered photosynthetic hosts, such as plants and algae. The inefficiency of RuBisCO and promiscuity towards oxygen have directed efforts in protein engineering towards the generation of optimised mutants that overcome these limitations [74–75]. Though these studies have resulted in the recombinant expression of RuBisCO in useful hosts such as *E. coli* [75], development of improved selection systems for directed evolution [74], and further elucidation of RuBisCO properties [76], little progress has been made toward expression of an enzyme which is more efficient and less promiscuous. A possible explanation for this was provided by Tlusty, Milo and coworkers [77]. By processing kinetic data from various RuBisCO enzymes, it was found that variations in enzyme specificity and velocity are mutually constrained. Within this limited space, it appears that the various wild-type enzymes have been optimised through evolution to operate within their respective environments. Point-mutations in the protein itself are therefore unable to lead to great improvements in enzyme efficiency. A more promising strategy may be to employ outlying natural variants of RuBisCO that display the best properties, such as those from red algae, in combination with other components of the Calvin cycle carbon assimilation mechanism [39]. Long et al. [78] estimated that incorporation of wild-type enzymes, with higher CO_2_ specificity or higher catalytic activity, into C_3_ plants could potentially raise crop yields by more than 25%. Furthermore, incorporation of cyanobacterial carbon concentration mechanisms such as carboxysomes, combined with RuBisCO variants adapted to higher CO_2_ concentrations, could result in a 36% to 60% crop yield increase [79].

The main difficulties of heterologous expression of RuBisCO for CO_2_ fixation relate to the poorly understood post-translational steps for production of the fully active enzyme that require the action of specific chaperones as well as a separate enzymatic species, RuBisCO activase. In some cases these have to also be incorporated into the host organism in order to obtain an active enzyme.

Recently there have been two important breakthroughs on carbon assimilation in plants through RuBisCO, relating to alternative components of the RuBisCO catalytic system. Whitney et al. [80] increased the expression levels of a heterologous RuBisCO in tobacco plants, through co-expression of a RuBisCO chaperone that facilitates the assembly of the active multimeric enzyme. This resulted in two-fold increases in CO_2_ assimilation rate and plant growth. Hanson, Parry and co-workers [39] were able to prepare tobacco plants that expressed cyanobacterial RuBisCO together with a protein that forms part of the carboxysomal structure, which led to the generation of macromolecular complexes that are observed early in the carboxysomal biogenesis in cyanobacteria. In addition, the engineered plants were photosynthetically active, and the RuBisCO complex showed higher specific activities than the enzyme in the control tobacco line.

Algae that utilise efficient variants of RuBisCO for fixation of CO_2_ have been targeted as a biomass source for a third generation of biofuels, due to their lack of requirement for arable land [81]. In addition, microalgae have been employed for the production of chemicals as a metabolic end-product of the fixed carbon, with particular emphasis on oils for use as biodiesel feedstock [82]. Cyanobacteria, mainly *Synechocystis* spp., have proven easier to engineer than their algal and plant counterparts and have also been applied to generate higher titres of oils and alcohols [18].

**Figure 3:**
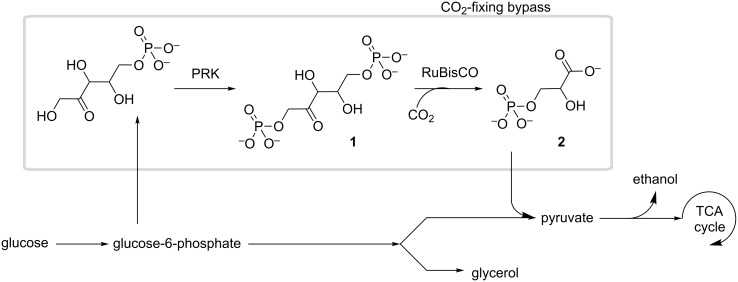
RuBisCO CO_2_-fixing bypass installed in *E. coli* and *S. cerevisiae* to increase carbon flux toward products of interest. PRK: phosphoribulosekinase.

Despite difficulties related to heterologous expression, recently there have also been reports of succesful use of RuBisCO in non-photosynthetic host organisms (Figure 3). In *E. coli* it was possible to incorporate a CO_2_-fixing bypass in central metabolism through expression of phosphoribulosekinase to produce ribulose-1,5-bisphosphate (**1**) (Scheme 1), and a cyanobacterial RuBisCO along with a RuBisCO-folding chaperone from the same source [21]. It was found that the main limiting factor to carbon fixation was the availability of CO_2_ in *E. coli*, and the yield could be increased through incorporation of a cyanobacterial carbonic anhydrase. In the yeast *Saccharomyces cerevisiae*, spinach phosphoribulosekinase was able to provide the bisphosphate **1** to a prokaryotic RuBisCO from *Hydrogenovibrio marinus*, which folded with the aid of *E. coli* protein chaperones (GroEL/GroES) [83]. This resulted in catalysis of CO_2_ fixation and increase of carbon flux towards the ethanol product and away from glycerol, a major fermentation byproduct (Figure 3).

#### Synthesis of dicarboxylates through pyruvate carboxylation

**Scheme 5:**
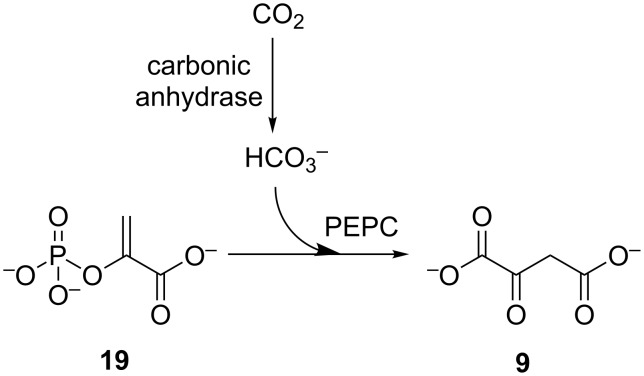
Integrated biocatalytic system for carboxylation of phosphoenolpyruvate (**19**), using PEPC and carbonic anhydrase.

Enzymatic carboxylation of a pyruvate backbone offers an avenue to dicarboxylates, which are important biotechnological targets, through the use of CO_2_ as feedstock. As seen, this may be carried out by pyruvate carboxylase or PEPC which acts on phosphoenolpyruvate (**19**). Purified PEPC has been used in an integrated system with carbonic anhydrase for in vitro carbon capture and transformation to oxaloacetate (**9**) (Scheme 5) [84]. This system has been further optimised with engineered variants of PEPC leading to increased rates and yields of CO_2_ transformation [85].

**Scheme 6:**
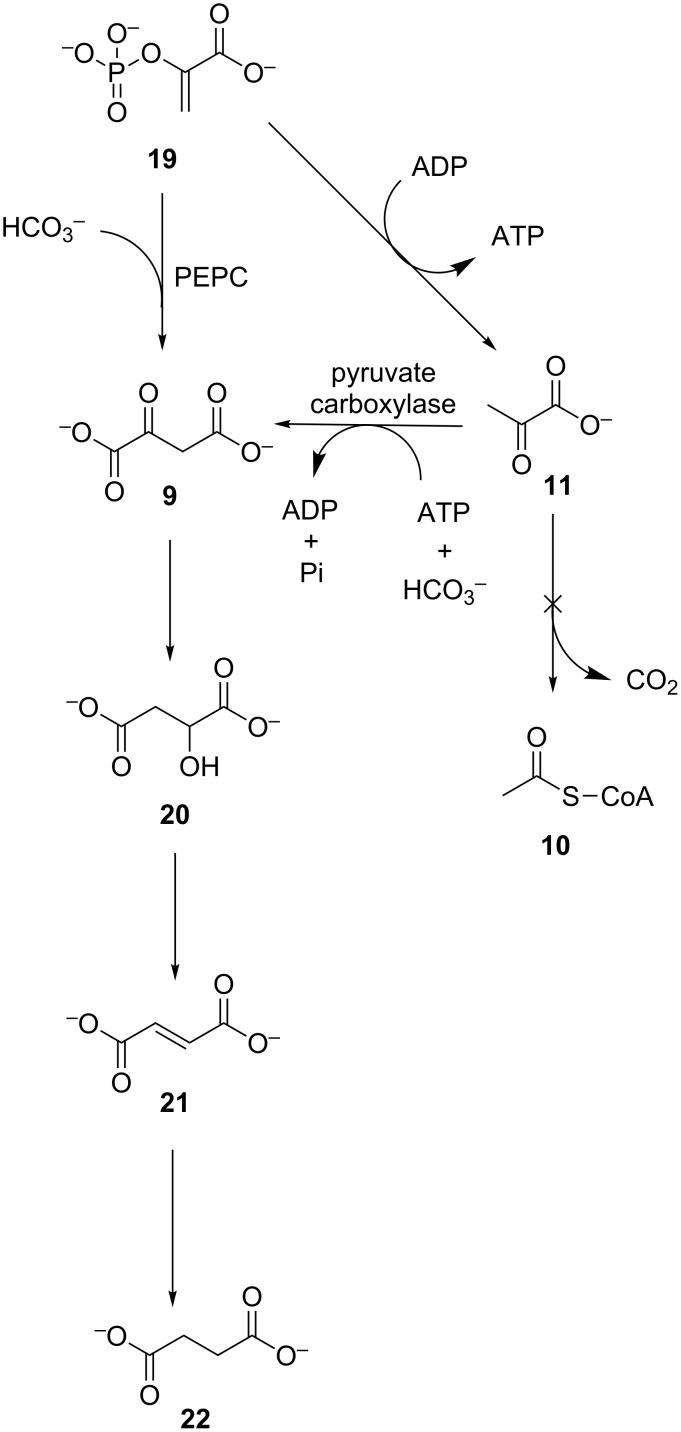
PEPC and pyruvate carboxylase catalysed carboxylation of pyruvate backbone for the generation of oxaloacetate (**9**) and other dicarboxylates.

In *E. coli* fermentative processes, as presented in Scheme 6, PEPC is used to produce oxaloacetate (**9**) directly from phosphoenolpyruvate (**19**) from glycolysis, through carboxylation with HCO_3_^−^. This may then be further transformed, by reversal of the activity of native oxidative TCA cycle enzymes, to produce malate (**20**), fumarate (**21**) and succinate (**22**), all of which have been listed in the top twelve target platform chemicals from biomass, by the US Department of Energy [86]. In this way, overexpression of *Sorghum vulgare* PEPC in *E. coli* resulted in higher fermentative yields of succinate (**22**) [87]. Recombinant co-expression of cyanobacterial carbonic anhydrase in *E. coli* BL21(DE3) increased available HCO_3_^−^ resulting in a higher than five-fold increase in the observed activity of endogenous PEPC [88]. Similarly, strains with overexpressed PEPC have been engineered for the production of high yields of fumaric acid (**21**) [89].

As some phosphoenolpyruvate (**19**) is lost to pyruvate (**11**), pyruvate carboxylase, not present naturally in *E. coli*, was used to increase the carbon flux to the desired products, by providing a secondary oxaloacetate (**9**) production route through CO_2_-fixation. In this way, *E. coli* strains overexpressing pyruvate carboxylase have been applied to CO_2_ fixation with the production of equimolar succinate (**22**) [90]. In addition the succinate yields were found to strongly depend on CO_2_ availability and increased by up to four-fold under increased CO_2_ partial pressures. Such engineered *E. coli* strains were also able to utilise CO_2_ generated during ethanol fermentation with *Saccharomyces cerevisiae* as the substrate for succinate production, through an integrated bioprocess [91]. Through gene deletion, other undesirable pyruvate consumption reactions such as lysis to acetyl-CoA (**10**) with liberation of CO_2_ could be blocked, allowing improved yields of dicarboxylates [92]. The carbon from CO_2_ was also directed to other products through the use of other types of host organisms. Overexpression of *E. coli* PEPC in *Propionibacteria* resulted in increased rates of propionic acid production as well as increased rates of carbon fixation under higher CO_2_ partial pressures [93–94].

#### Acyl-CoA carboxylases

Though acetyl-CoA carboxylases are widely distributed in living organisms, the existence of bifunctional variants with a role in autotrophy has attracted further interest for their biotechnological applications in CO_2_ transformation technologies. The autotrophic enzymes from *Metallosphaera sedula* and *Acidianus berleyi* have been purified and found to be catalytically active in vitro for the production of malonyl-CoA through acetyl-CoA carboxylation [71,95]. As seen in Scheme 4, two subsequent steps in the 3-hydroxypropionate/4-hydroxybutyrate cycle lead to further reduction of the fixed carbon for the generation of 3-hydroxypropionate (**16**), a platform chemical also in the US Department of Energy top twelve [86,96]. Archaeal thermoacidophilic *Metallosphaera sedula* genes were utilised in the hyperthermophilic archaeon *Pyrococcus furiosus* to express the first three steps of the autotrophic 3-hydroxypropionic/4-hydroxybutyrate cycle for the synthesis of 3-hydroxypropionate (**16**) [97–98]. This was carried out at 70 °C, where the *Metallosphaera* enzymes show optimal activity and background metabolism of *Pyrococcus furiosus* does not interfere.

#### Decarboxylases

**Scheme 7:**
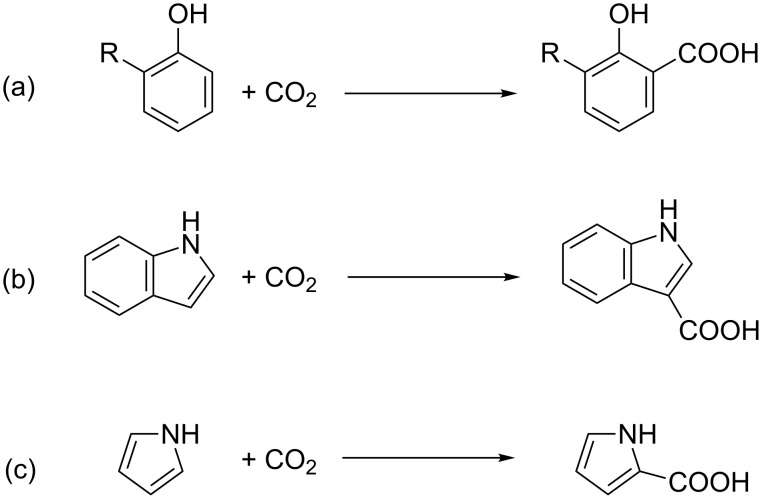
Decarboxylase catalysed carboxylation of (a) phenol derivatives, (b) indole and (c) pyrrole.

A number of enzymes are capable of catalysing the reversible interconversion of lipophilic aromatics and the more polar respective carboxylates [12]. It is thought these reactions may proceed in the carboxylation direction as a detoxification mechanism under anaerobic conditions, where oxidative degradation is not possible. In work pioneered by Nagasawa and coworkers [99–102], Kirimura and coworkers [103–104], and Faber and coworkers [105–109], these enzymes have been successfully applied in vitro under conditions that drive the equilibrium toward carboxylation, such as high CO_2_ concentration. Successful examples include the carboxylation of phenol and hydroxystyrene derivatives including catechol [102], guaiacol [110], indole [101] and pyrrole [100] (Scheme 7).

#### Isocitrate dehydrogenase

As discussed above, as part of the reductive TCA cycle (Scheme 2) isocitrate dehydrogenase catalyses the carboxylation of 2-oxoglutarate (**6**) to produce isocitrate (**7**). Exploitation in biotechnological applications has been challenging due to the unfavourable thermodynamics of the carboxylation. Recently, the use of purified isocitrate dehydrogenase for CO_2_ fixation was reported [111]. Carbon fixation was driven thermodynamically by maintaining a low pH, where CO_2_ concentrations are highest, and coupling the reaction to aconitase catalysed removal of isocitrate (**7**) to produce aconitate. Switching the pH allowed for subsequent release of the captured CO_2_ and regeneration of the carbon-capture substrate 2-oxoglutarate (**6**). Though this application is aimed at CO_2_ sequestration rather than transformation, it shows that the reductive TCA cycle isocitrate dehydrogenase may be used in vitro to fix CO_2_ to a species that may be further transformed enzymatically.

#### FDH catalysed formate production

Due to the direct CO_2_ reduction to a C_1_ species, as opposed to carboxylation of a secondary substrate catalysed by most other enzymes, FDHs have attracted more widespread attention as catalysts for the transformation of CO_2_ with numerous examples in recent literature. Applications span all aspects of enzyme technology including isolated biocatalysts, immobilised biocatalysts, whole-cell catalysts and bioelectrocatalytic systems. Theoretical studies modelling potential formatotrophic organisms showed significant promise for such systems [112].

**Isolated FDH.** Enzymes from acetogenic sources have been characterised and found to be capable of catalysing CO_2_ reduction in vitro under thermodynamically favourable conditions. Acetogenic FDH from *Clostridium thermoaceticum* (now *Moorella thermoacetica*) was reported by Wood and Ljundahl in 1966 [113], where an exchange between ^14^CO_2_ and formate was observed, though no net synthesis of formate. Thauer [114] was the first to observe a net CO_2_ reduction to formate for the acetogenic FDH by recycling of the reduced cofactor, and prove that this enzyme utilised NADPH for the reduction of CO_2_ as the first step in one branch of the Wood–Ljungdahl pathway. Similarly, FDH in cell-free lysate of *Clostridium acidiurici* catalysed CO_2_ reduction to formate with reduced ferredoxin and NADH [115]. Earlier, it had been shown that it was possible for an enzyme found in the related non-acetogenic *Clostridium pasteurianum* to carry out direct reduction of CO_2_ to formate with reduced ferredoxin alone, rather than through a two-step process involving acetyl-CoA as a CO_2_ acceptor, disproving the established view at that time that biological CO_2_ reduction may only proceed indirectly [116]. Furthermore, Thauer et al. [117] were able to prove that this FDH utilises CO_2_, rather than HCO_3_^−^, as the active species, through experiments carried out at low temperatures where CO_2_ hydration is slow. The enzyme from *Clostridium carboxidivorans* was recombinantly expressed in *E. coli* and shown to display higher CO_2_ reducing activity and poorer affinity for formate, as compared to a non-acetogenic *Candida boidinii* FDH prepared in parallel, known to efficiently oxidise formate [62]. This suggests that weak formate binding contributes toward the catalytic preference of the acetogenic enzyme. *Clostridium autoethanogenum* was purified as a complex with an electron bifurcating hydrogenase that is NADPH and ferredoxin dependent, and found to catalyse reduction of CO_2_ with NADPH and reduced ferredoxin or H_2_ [63]. An FDH was also purified as a complex with hydrogenase from the acetogen *Acetobacterium woodii* and found to directly utilise H_2_ as an electron donor for the reduction of CO_2_ [118].

Furthermore, there is a growing list of examples of non-acetogenic metallo-FDHs, naturally catalysing formate oxidation, found to also be capable of catalysing CO_2_ reduction in vitro. FDH from *Pseudomonas oxalaticus* was the first isolated enzyme reported to catalyse both formate oxidation and CO_2_ reduction under appropriate conditions, using substrate amounts of NAD^+^/NADH [119]. This enzyme was later used in the seminal work of Parkinson and Weaver [120], where electrons were supplied through a semiconductor photoelectrode using light in the visible spectrum (>1.35 eV) and coupled to FDH activity through a mediator to drive CO_2_ reduction. Two W-dependent FDHs, isolated from the syntrophic bacterium *Syntrophobacter fumaroxidans*, showed high catalytic activity for CO_2_ reduction, using reduced methyl viologen as the electron donor. Later, one of these was immobilised onto an electrode and shown to reduce CO_2_ electrochemically through direct use of the electrons provided [121]. In this way the reaction could be electrochemically driven in either direction. Recently the Mo-dependent FDH from *E. coli* was shown to be capable of catalysing CO_2_ reduction employing a similar approach [122]. An oxygen-tolerant Mo-dependent FDH from *Rhodobacter capsulatus* was reported to catalyse the reduction of CO_2_ with NADH [123].

FDHs without metal cofactors have also been employed to reduce CO_2_ in vitro. Despite interest in application of *Candida boidinii* FDH due to its stability, the observed turnover for this enzyme is generally low. However, application of a bioelectrochemical system allowed production of formate from CO_2_ with proton transfer from an electrical source through NAD^+^ to this FDH [124]. Choe et al. [125–126] showed that a series of robust acidophilic nonmetallo-FDHs were particularly useful in the catalysis of CO_2_ reduction. As these enzymes are stable at the lower pH ranges where the concentration of solvated CO_2_ is highest, improved formate yields were obtained.

**Figure 4:**
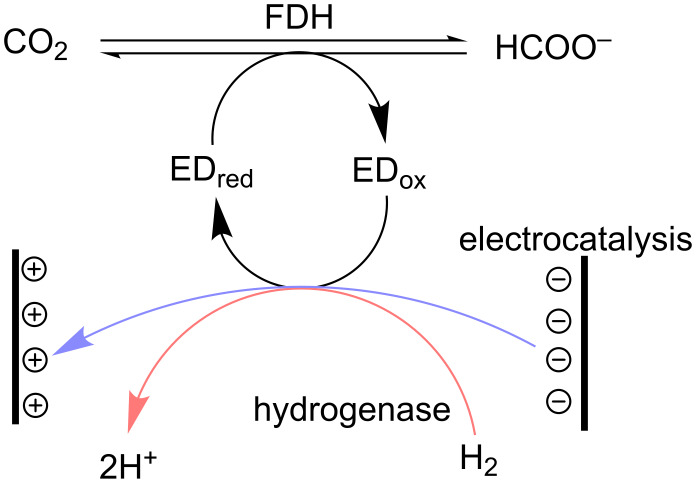
Formate dehydrogenase (FDH) catalysed reversible reduction of CO_2_ to formate with electron donor regeneration through hydrogenase-catalysed H_2_ oxidation (red arrow) or electrochemical reduction at a cathode (blue arrow).

All this suggests that the ability of FDHs to reversibly catalyse formate and CO_2_ interconversion is broadly distributed in nature, irrespective of metabolic directionality, however, catalytic properties vary greatly depending on the source organism. As expected, the enzymes that naturally catalyse CO_2_ reduction and highly homologous FDHs from formate oxidation pathways display higher reduction activities than FDHs of lower homology. The possibility of recycling the reduced electron donating cofactor, through the action of a second enzyme such as hydrogenase, or through direct or mediated electron delivery from an electrode, greatly enhance the potential of FDHs for application in large-scale biotechnological processes (Figure 4).

**Whole-cell FDH application.** In addition to the examples already mentioned for in vitro enzymatic production of formate, this has also been achieved using resting or immobilised cells as catalysts. Due to the ease of combination of multiple enzymatic activities in whole-cell applications, there has been a particular focus on coupling FDH activity to a hydrogenase, with which it is commonly found as the formate hydrogen lyase complex in nature. To this effect, immobilised *Alcaligenes eutrophus* (reclassified as *Ralstonia eutropha*) whole-cell catalysts were able to catalyse hydrogenation of CO_2_ to similar levels as Pd adsorbed on activated carbon [127]. In the previously mentioned work [118] on the purified acetogenic FDH-hydrogenase complex from *Acetobacterium woodii*, a whole-cell biocatalyst was also reported, generating high yields of formate from CO_2_ and H_2_. Resting cells from the common biotechnological host *E. coli* have been known to generate modest yields of formate from CO_2_ hydrogenation, when grown on formate for induction of the native enzymes [128]. More recently, by overexpressing suitable recombinant FDHs in *E. coli* JM109(DE3), high formate yields were obtained from CO_2_ hydrogenation, without need for cellular growth on formate for induction [129]. An alternative whole-cell system was later reported, using an electrochemical cell, where the reducing equivalents are generated by an electrode, rather than H_2_ oxidation, as has been done for purified enzymes [130].

**Figure 5:**
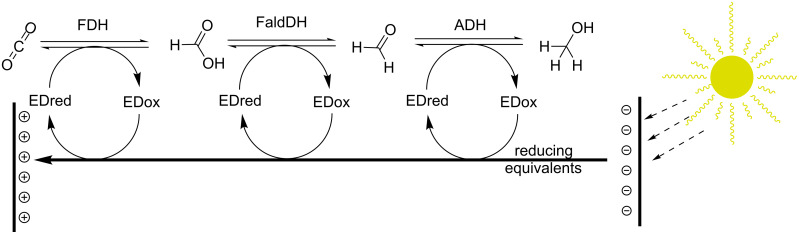
Sequential generation of formate, formaldehyde and methanol from CO_2_ using reducing equivalents sourced through electrochemical cells or photocatalysts. ED: electron donor, FDH: formate dehydrogenase, FladDH: formaldehyde dehydrogenase, ADH: alcohol dehydrogenase.

**Methanol production through formate.** Due to the advantages of direct formatogenesis from CO_2_, there has been a number of investigations into further biocatalytic conversion of formate into other desirable chemicals. A possibility that has gathered much attention is the consecutive reduction to formaldehyde and methanol, first described by Kuwabata and co-workers [131–132]. This is of particular interest due to the potential use of methanol as a fuel. Methanol production has been achieved in vitro utilising FDH in series with formaldehyde dehydrogenase (FaldDH) and alcohol dehydrogenase (ADH) [133–136]. One of the main hurdles to the utilisation of this process relates to the requirement for the additional two enzymes to work in the reverse to physiological direction, as well as the generally unfavourable thermodynamic equilibria. An attractive approach utilised photocatalysts to generate electrons from solar energy, which in turn were donated for the production of methanol [137]. Though methanol yields and catalyst efficiencies are low, these results are highly promising for the future development of biochemical systems for the solar-driven generation of formate, formaldehyde and methanol from CO_2_ (Figure 5).

**FDHs for hydrogen storage.** The significance of biocatalytic systems for the production of formate with reducing equivalents from H_2_ extends beyond the generation of a platform chemical. Formate has also been targeted as a form of chemical storage of hydrogen fuel, due to energetic demands and hazards associated with H_2_ liquefaction, transport and storage [138–139]. Effective use of CO_2_ to store H_2_ would enable a sustainable hydrogen based economy, through carbon neutral technologies. Formate in particular, due to its chemical properties and the atom efficiency in complete stoichiometric retention of hydrogen, has been touted as a very promising reduced form of CO_2_ [138,140–141]. Consequently, many catalytic systems working in the reverse direction have also been investigated, for the regeneration of H_2_, along with CO_2_.

**Figure 6:**
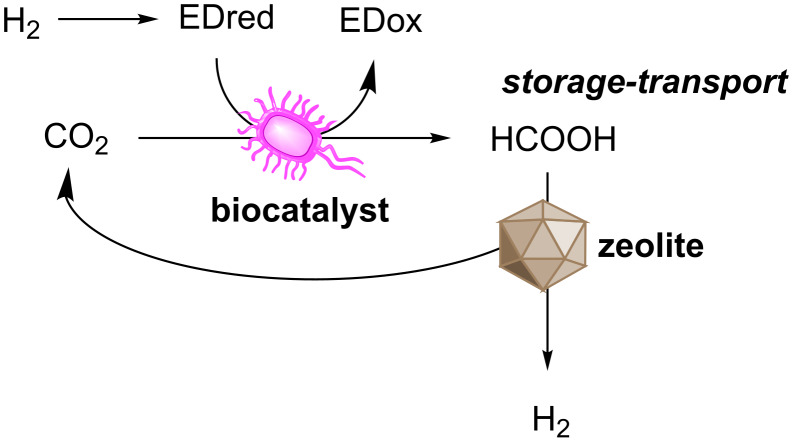
Hydrogen storage as formic acid through biocatalytic hydrogenation of CO_2_ and subsequent on-demand release through zeolite catalysed dehydrogenation.

Many organisms, including *E. coli*, naturally produce H_2_ as an electron sink for oxidative pathways [142]. As a result, whole-cell systems have been described that work efficiently toward formate oxidation and direct electron delivery to a hydrogenase [143–144]. However, biocatalytic systems are unlikely to become suitable for decentralised H_2_ release, as for example will be required in hydrogen fuelled transportation vehicles. Transition metal catalysts have been reported to reach desired turnovers [138], however, in these cases cost and metal availability become hurdles in sustaining a hydrogen economy. Zeolite systems utilising Ge or Si, recently described, were able to efficiently dehydrogenate formic acid. This was guided through computational calculations, allowing the design of a zeolite catalyst displaying over 94% selectivity over the counter-productive formate dehydration reaction [145]. The combination of biological systems for centralised hydrogen storage through CO_2_ reduction as formate, with cheap zeolite catalysts for decentralised on demand hydrogen regeneration appears a very promising sustainable approach toward a hydrogen economy (Figure 6).

#### In vitro production of CO with CODH

Reduction of CO_2_ to CO through in vitro application of CODH has been of interest as the enzymatic product may be further converted into hydrocarbons through the Fischer–Tropsch process [146]. In work carried out by Armstrong, Ragsdale and coworkers [147–148], metal oxide nanoparticles were functionalised with CODH and photosensitised with a Ru dye to catalyse the reduction of CO_2_ using visible light. Further to this, the reported ability of a V-dependent nitrogenase to slowly reduce CO to various small-chain hydrocarbons holds much promise for the development of enzymatic processes to further transform CO into products of interest [149].

### Prospects and challenges for future biotechnological applications

In order for CO_2_ biotransformation to target a broad range of commodity chemicals, the CO_2_-fixing enzymes must be used as part of multi-enzymatic cascades that convert CO_2_ through multiple steps [111,150]. Such reactions may be performed in vitro, where the relative amounts of each biocatalyst and the intermediate concentrations during the reaction can be closely monitored and controlled. However, this is accompanied by a requirement in cost related to enzyme purification, proportional to the number of enzymes used. The application of enzymes within whole-cells allows their production and utilisation with minimal processing and circumvents biocatalyst purification, though in this case there are limitations related to substrate/product diffusion and background metabolic activity. The optimal approach in each case, as for any multi-enzymatic synthesis, will depend on a combination of factors such as the number of enzymes to be utilised, the ease of substrate and product diffusion through the cell membrane, and the presence of unwanted background reactions.

Within a well-understood cellular chassis, the heterologous expression of a CO_2_ fixing enzyme allows its use as a module that may be matched with other modules of choice, for the assembly of synthetic pathways [150–151]. In a CO_2_ transforming modular process, the CO_2_ fixing modules will play a central role, much like CO_2_ fixing enzymes do in a carbon assimilation pathway. However, the assembly will also include other genes that allow process control or express desirable features such as acid tolerance [152–153]. For these modules to be easily applied, the enzymes must be easy to express in heterologous hosts. This is greatly complicated by requirements for specialised cofactors or maturation and folding processes.

RuBisCO presents significant challenges for use in modular synthetic biology approaches, due to the observed inefficiency and requirement for expression of large amounts of protein. The difficulty of expression in hosts that do not naturally contain RuBisCO, such as *E. coli*, and the complicated nature of the heterologous RuBisCO systems currently developed in transgenic plants [80], means that the Calvin cycle is a challenging target for synthetic biology in non-photosynthetic microorganisms. Efforts focusing on increasing carbon fixation yields through optimisation of RuBisCO expression and activity may lead to optimised plant based synthetic systems [18,40].

In microbial systems carboxylases are promising candidates for modular design, due to their broad distribution in living organisms and lack of particular requirements in cofactors. Also the great variety of carboxylases found in nature represents a very large library from which suitable modules may be sourced that introduce carbon into metabolic pathways [26,154]. On the other hand, in order to generate a synthetic pathway where the only carbon input is CO_2_, these enzymes would also require the co-expression of cyclic pathways to recycle the co-substrates that are carboxylated. This may greatly hinder the overall process, as the metabolic pathways that have been developed by nature to carry out these tasks contain many steps and a number of unfavourable reactions. Indeed, attempts to transfer entire autotrophic CO_2_ fixation pathways into *E. coli* have been unsuccesful [155].

Dehydrogenases used in the reductive acetyl-CoA pathway, do not present this complication as the CO_2_ is reduced directly to another species, either formate or CO, with no other reactant other than a source of electrons. This means that a single enzymatic module is able to catalyse the incorporation of CO_2_ as a C_1_ species, with no other carbon requirement. In this case, the difficulties associated with expression of enzymes from niche organisms in heterologous hosts, such as requirement for particular metal cofactors and oxygen stability, complicate use in modular approaches. Also, the metabolic product must be efficiently transformed into other species in order to drive this energetically uphill carbon fixation process. Finally, as formate and CO are not metabolites in central anabolic pathways, it may be challenging to find suitable pathways that allow access to the variety of chemicals that may be produced through metabolism. This will inevitably require heterologous expression of the full reductive pathway, for production of acetyl-CoA, which however is extremely challenging due to the requirement for use of poorly understood enzymes and unusual cofactors. A recent breakthrough came with the production of a computationally designed enzyme, catalysing the carboligation of three formaldehyde units into dihydroxyacetone, thus providing direct access to central carbon metabolism through formate [156].

**Figure 7:**
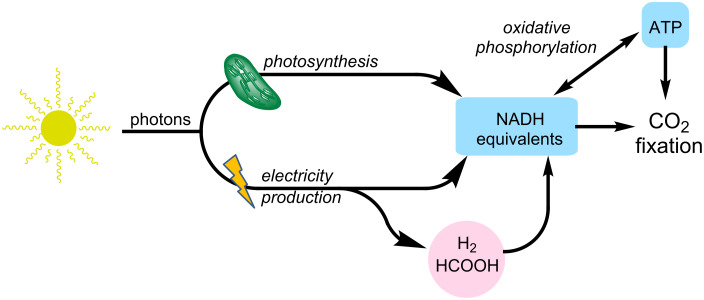
Schematic showing required flow of reducing equivalents for CO_2_ fixation through biotechnological applications.

**Sourcing of reducing equivalents.** As mentioned above, any process that transforms CO_2_ into other chemicals, where the carbon is in a more reduced state, represents a net reduction. Therefore there is a requirement for reductive potential in the form of electrons, and the method used to source these will greatly define the utility of the overall process (Figure 7). The ATP required to drive CO_2_ fixation processes within living systems will be mainly produced using reducing equivalents through the complicated mechanism of oxidative phosphorylation.

Ultimately the most sustainable source of reducing equivalents is sunlight [20]. Solar energy may be directly utilised through the application of photosynthetic machinery employed by photoautotrophs to carry out the “light reactions” of photosynthesis. This will require technological advances, such as the development of bioreactors capable of maximising exposure to sunlight [157]. Another limitation to any approach relying on photosynthesis to harvest solar energy is the inherently poor efficiency and sensitivity of photosynthetic pigments and reaction centres, as highlighted by Michel [158]. An alternative approach is to convert solar energy into electricity for use as a source of electrons [20,159]. As seen, a number of enzymes and organisms are indeed capable of directly accepting electrons from electrodes in bioelectrochemical systems [160–162]. The use of electricity generated through photovoltaics allows the mediated application of solar energy for the fixation of CO_2_. Finally, electrons can be stored within chemical species that may then be oxidised by organisms to regenerate the electrons on-demand [20]. Hydrogen and formic acid appear most suited for such applications, due to their chemical properties, and the existence of efficient biological tools for electron regeneration through oxidation.

## Conclusion

It is evident that the use of biological catalysts for CO_2_ fixation and conversion to a variety of chemicals is a promising approach, not limited by the availability of natural enzymes. However, in order for these to be employed in suitable bioprocesses, where they may be assembled into multi-enzymatic synthetic cascades, suitable methodologies for facile recombinant expression need to be developed further. This will extend beyond simple expression of a single gene, and may require simultaneous expression of multiple subunits, expression of seleno-proteins, proteins that deliver particular cofactors, as well as chaperones and maturation proteins that allow the production of the final active biocatalyst. Furthermore, the various biological mechanisms used in nature to improve the activity of these enzymes must be fully understood, in order to be suitably harnessed for application in synthetic processes. Host organisms must be developed with features geared towards the fixation of CO_2_ and its transformation through multiple enzymatic steps. Finally the reducing equivalents required for the carbon fixation step, as well as subsequent transformations, must be harnessed efficiently. Suitable technological platforms are yet to be developed.

Though there is much progress to be made before CO_2_ fixing enzymes may be readily used as modules in designer synthetic pathways, the rapid progress that is being made in the fields of genetic engineering, bioinformatics and synthetic biology, as well as renewable electricity generation and bioelectrochemical engineering hold much promise for the development of the biotechnological platforms that will support a future carbon bio-economy.
